# Effectiveness of referral to a population-level telephone coaching service for improving health risk behaviours in people with a mental health condition: a randomised controlled trial

**DOI:** 10.1186/s12889-025-21614-w

**Published:** 2025-02-19

**Authors:** Grace Hanly, Elizabeth Campbell, Kate Bartlem, Julia Dray, Caitlin Fehily, Kim Colyvas, Tahlia Reynolds, Sandy Davidson, Sarah Yeun-Sim Jeong, John Wiggers, Luke Wolfenden, Jenny Bowman

**Affiliations:** 1 School of Psychological Sciences, University of Newcastle, Callaghan, NSW Australia; 2 Population Health Research Program, Hunter Medical Research Institute, New Lambton Heights, NSW Australia; 3 Hunter New England Population Health, Wallsend, NSW Australia; 4 Centre for Population Health, NSW Ministry of Health, St Leonards, NSW Australia; 5 Centre for Mental Health Research, National Centre for Epidemiology and Population Health, The Australian National University, Acton, ACT Australia; 6 Susan Wakil School of Nursing and Midwifery, The University of Sydney, Sydney, NSW Australia

**Keywords:** Health behaviour, Mental health, Chronic disease, Exercise, Diet, Preventive care, Telephone coaching, Behaviour change

## Abstract

**Background:**

Telephone support services are a viable means of providing population-level support to reduce health risk behaviours. While research exists on the effectiveness of Quitlines to reduce smoking, there is limited other research investigating whether telephone services can provide effective behaviour change support for people with a mental health condition for behaviours including physical activity, healthy eating, and weight management. The aims of this trial were to evaluate the effectiveness of referral of people with a mental health condition to a population-level telephone coaching service to improve health risk behaviours and increase attempts to do so.

**Methods:**

A parallel-group randomised controlled trial was conducted. Participants with a mental health condition (*N* = 681) were assigned to a control (health information pack) or intervention group (information pack and referral by the research team to a coaching program). Data were collected via telephone surveys at baseline and six months post-recruitment. Primary outcomes were: (1) weekly minutes of moderate-to-vigorous physical activity, (2) daily fruit serves, (3) daily vegetable serves, and (4) attempted behaviour change/weight loss (yes/no; composite measure). Secondary outcomes included weight, Body Mass Index (BMI), and attempts to change each health behaviour individually.

**Results:**

Intention-to-treat analyses found no significant differential change between groups from baseline to six months for primary or secondary outcomes. By follow-up, 242/549 (44%) of intervention participants had enrolled in coaching and completed at least one call, with 16/242 having completed the program, 79 ongoing, and 147 withdrawn. Per-protocol analyses found attempting to improve at least one health behaviour/lose weight was significantly greater in enrolees (OR = 3.7, 95% CI 1.03—13.23) than the control group.

**Conclusions:**

Referral to the program did not improve risk behaviours or weight/BMI but did support behaviour change attempts. Contributing factors may include low program completion by follow-up and impact of COVID-19. Further research is required to better understand participation in and benefits of telephone coaching services for people with a mental health condition.

**Trial registration:**

Registered retrospectively with the Australian New Zealand Clinical Trials Registry (ACTRN12620000351910).

**Supplementary Information:**

The online version contains supplementary material available at 10.1186/s12889-025-21614-w.

## Background

The reduced life expectancy of 10–20 years experienced by people with a mental health condition in comparison to the general population [1–3] is mainly attributed to a higher burden from chronic diseases [3–7]; in turn related to a high prevalence of risk factors including obesity [8] and health risk behaviours [8–14] (tobacco smoking [9–12], harmful alcohol consumption [8], poor nutrition [13], and inadequate physical activity [14]). Addressing this inequity is a policy priority, internationally [15, 16].

Telephone-delivered coaching can provide evidence-based behaviour change support with broad population reach [17, 18] and overcome barriers to in-person support including lack of services or transport, and travel costs [17, 18]. Such barriers are especially relevant to people with a mental health condition, with high rates of socioeconomic disadvantage, suggesting that telephone coaching services may represent a valuable referral option for mental health services to support clients with lifestyle change. With respect to Quitline telephone counselling, people with a mental health condition have been shown to make up a significant proportion of users (40–46%) [19–21], and to gain effective support for quit attempts and smoking cessation [19, 22–24].

For behaviours such as physical inactivity and poor nutrition, systematic review evidence indicates that telephone interventions can be effective for the general population [25]. Research trials for people with a mental health condition specifically, however, are limited in number: two pre-post evaluations [26, 27] and two randomised controlled trials (a pilot with *n* = 22 participants in the United States [28] and an Australian feasibility trial with *n* = 43 particpants [29]) were identified. All studies reported some positive impact including improved physical activity [28, 29], fruit and vegetable intake [27], overall diet quality [27], reduced sitting time [27], and weight [26]. Research investigating the effectiveness of telephone coaching services delivered at a population-level to the general population or to people with a mental health condition, for such behaviours is limited [1, 18, 26]. One such service is the New South Wales (NSW) Get Healthy Coaching and Information Service® (GHS); a government-funded Australian service that provides free telephone coaching to support healthy eating, physical activity, and weight management and to reduce alcohol consumption. Two pre-post studies have reported positive impacts of the GHS for people with [30] and without [18, 30] mental health conditions on aspects of nutrition, physical activity, weight, and Body Mass Index (BMI), for those who completed the coaching program [18, 30]. In the largest of these (5,629 GHS participants) [30], Bradley et al. (2021) found that participants with a mental health condition represented 33% of coaching clients and experienced positive outcomes upon completion, though less comprehensively so than those without a mental health condition. [30] Clients without a mental health condition made significant improvements in all eight outcomes examined (related to weight, physical activity, and nutrition), those with a mental health condition demonstrated improvements in six (excepting waist circumference and vigorous physical activity). In terms of magnitude of change, clients without a mental health condition demonstrated greater improvements to their weight and vegetable intake, with no differences identified between the groups for other outcomes [30]. However, only 31% of all enrolees with a mental health condition completed the program (compared to 36% of those without), with most deciding not to continue or being lost to follow-up by the GHS. Studies utilising more rigorous designs are needed to provide greater insight into the potential population-level impact that may result from mental health service clients being referred to such a service.

This study assessed the effectiveness of referral to a population-level telephone coaching service (the GHS) to increase: (1) physical activity (2) fruit consumption (3) vegetable consumption, and (4) attempts to improve health behaviours or lose weight for people with a mental health condition (primary outcomes). Secondary outcomes including self-reported weight and BMI were assessed.

## Methods

### Study design and setting

**Fig. 1:**
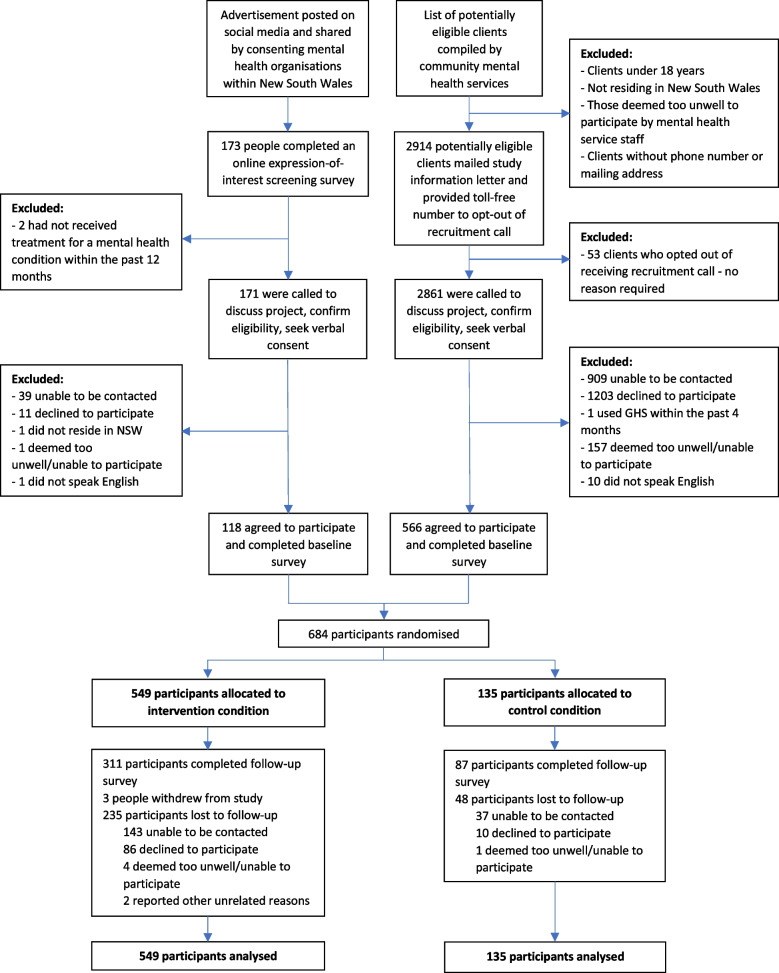
Study design flow diagram

A parallel group randomised controlled trial was conducted in NSW, Australia; October 2019 to July 2021 (Fig. 1). The study was approved by ethics committees (HNE HREC 2018/ETH00377; UoN HREC approval No. H-2019–0013) and registered retrospectively (ACTRN12620000351910). Trial was designed and is reported in accordance with CONSORT guidelines [31]. Further details are in the study protocol [32].

Clients were recruited from ten government community mental health services (CMHS) across Hunter New England and Central Coast health districts in NSW (October 2019 to January 2021), with additional social media recruitment strategies introduced in May 2020. Participants were randomly assigned to either the intervention (health information brochures and referred to GHS by the researchers) or control condition (brochures only) in a 4:1 ratio [32].

### Participants and recruitment

Eligibility criteria included: i) 18 years or older ii) NSW resident iii) self-report of a diagnosed mental health condition and having received mental health care within 12 months iv) a telephone, and v) willingness for contact by a telephone support service. Exclusion criteria included: i) deemed too unwell/clinically inappropriate to participate by CMHS staff ii) no contact details, or iii) reported use of GHS within four months [32].

#### Community mental health service clients

Eight CMHSs provided a list of all potentially eligible clients. Each week, a random sample of up to 100 clients were mailed a study information statement from their mental health service. Clients from two further CMHSs who had participated in a previous project and expressed interest in research were mailed the study information. The statement provided a number to ‘opt-out’ of further contact about the study.

#### Social media expression of interest

To aid recruitment during COVID-19, a study advertisement was featured on Facebook and Twitter and shared by several mental health and research organisations via social media (in May 2020). The advertisement linked to a website with further information and a form to assess eligibility and collect contact details.

### Intervention

#### Control condition

Control participants were mailed a health information pack about health risk factors [32]. At six-month follow-up they were informed about GHS and provided details to self-refer.

#### Intervention condition

Intervention participants were mailed the same health information pack and were referred to GHS by the research team using the GHS online referral form. GHS followed standard service procedure to contact those referred to collect service baseline information and discuss enrolment in a coaching program. Coaching consisted of evidence-based support, using motivational interviewing and self-regulation principles, to assist clients to make healthy lifestyle changes [18] targeted to individual health goal/s. Those not electing to take part in coaching were offered a single ‘information only’ call about making healthy lifestyle changes.

Coaching participants were enrolled in either the standard program or a program tailored for a specific health concern (Type 2 Diabetes Prevention, Alcohol Reduction) or group (Aboriginal and Torres Strait Islander, Chinese, Pregnancy). The GHS program included up to 10 coaching calls (or 13 for tailored programs) over approximately six months; with a health coach with qualifications aligned to participants’ selected goal (e.g. exercise physiologist or dietician) and mental health first aid training. Calls were typically 15 min long and scheduled according to enrolees’ preferences.

Participants were considered to have completed the program if they completed all coaching calls or, if they completed at least four coaching calls and reached their health goal (early completion). Those who informed GHS they were withdrawing before completing all calls and without reaching their goal were coded as ‘active withdrawal’, while those who could not be contacted for a scheduled coaching call after four attempts (or six attempts for those who identified as Aboriginal or Torres Strait Islander) were coded as ‘passive withdrawal’.

To maximise the rate of successful GHS contact with referred participants, the research team attempted to contact those who GHS was unable to reach for the initial call. Two attempts were made by the research team to recontact these participants, and those subsequently consenting were re-referred.

### Data collection

Baseline and follow-up surveys were used to collect outcome data for this trial. Computer assisted telephone interviews (CATI) were conducted by experienced telephone interviewers and took approximately 30 min. The surveys were compiled for this study using previously used surveys and validated measures (referenced below) (see Additional File 1 for full survey of reported measures). Calls to confirm eligibility, gain consent and administer the baseline surveys occurred between October 2019–January 2021, when recruitment target was met and any ongoing contacts were finalised, and follow-up surveys six months post-recruitment April 2020–July 2021. A report was provided by GHS at the conclusion of the trial summarising contact with intervention group participants.

### Outcomes and measures

#### Participant characteristics

Demographic and clinical information (baseline only) included: age, gender identity, highest level of education, employment status, Aboriginal and/or Torres Strait Islander status, postcode (to calculate geographic remoteness [33] and socioeconomic index of disadvantage [34]) and primary mental health diagnosis (Table 1). Medications for mental health conditions were collected at baseline and follow-up.

#### Primary outcomes

(i) *Minutes of moderate to vigorous physical activity (MVPA) per week:*Measured using the Simple Physical Activity Questionnaire (SIMPAQ), validated for use by people with a mental health condition [35].
(ii) *Daily serves of fruit consumed:*A single-item; “over the past month… how many serves of fruit do you eat in a usual day?” (serves/don’t know) [8, 36].
(iii) *Daily serves of vegetables consumed:*a similar measure [8, 36].
(iv) *A composite measure of six items assessing recent attempts to improve any one of the following health behaviours, or lose weight (yes, no, don’t know):* “In the last four months, have you attempted to…” (i) increase physical activity (ii) increase fruit consumption (iii) increase vegetable consumption (iv) make any other changes to diet or nutrition (v) reduce alcohol consumption (for participants that reported consuming any alcohol), or (vi) lose weight (excluding those with an eating disorder).

#### Secondary outcomes

- *(i) Weight*: self-reported (kg, pounds, or stone).
- *(ii) BMI* (kg/m^2^)*:*self-reported height (cm or feet/inches)(baseline only) and weight [37].
- *(iii – viii) Any attempts to change each health behaviour, or weight:* Six items as described in primary outcomes, assessed individually.

#### Additional measures

The short form of the Alcohol Use Disorders Identification Test (AUDIT-C) [38, 39] was used to measure alcohol consumption, and participants were asked “how often did you smoke tobacco products in the last month?”. Sedentary hours/day were calculated from SIMPAQ. Psychological wellbeing was measured using the six-item Kessler-6 (K6) [40] and quality of life was assessed with the 12-item Assessment of Quality of Life instrument (AQoL-4D) [41]; both measures have been validated for a mental health population [42, 43].

#### Process measures

GHS provided service data for coaching enrolees from their date of referral to report extraction (29 October 2021) including: date of first contact; date of enrolment; program selection; health goal; total coaching calls; date of most recent coaching call; final program status (completion, early completion, active withdrawal, passive withdrawal, or still active); and date final status reached.

#### Acceptability and appropriateness of GHS

Short 4-item versions of the Acceptability of Intervention Measure (AIM) and the Intervention Appropriateness Measure (IAM) [44] were asked about the GHS for the intervention group at follow-up [44].

### Randomisation and blinding

An independent statistician generated a random allocation sequence for each recruitment site using permuted blocks of 5, 10, and 15 with a 4:1 allocation ratio for intervention and control respectively, which they uploaded to the randomisation feature of the data collection software where it could not be viewed or downloaded; once baseline data were collected, the system provided the allocation of each participant [32]. Participants could not be considered blind to their group as the CATI interviewers then told those allocated to intervention that they would be referred to GHS. For follow-up data collection, CATI staff were not aware of participants’ group allocation until the final survey section (which included questions about experience with GHS).

### Statistical methods

Analyses were conducted using SAS Version 9.4 [45] and assumed a statistical significance level of *p* ≤ 0.05.

Age was grouped into 10-year categories [46]. Postcode was used to calculate: Socio-Economic Index for Areas (SEIFA) categories dichotomised on the NSW median [34]; and remoteness using the Australian Statistical Geography Standard Remoteness Structure [33], dichotomised into ‘major cities’ or combined ‘inner/outer regional or remote’.

Descriptive statistics were used for participant characteristics at baseline, and process variables including intervention group contact with GHS, AIM and IAM. Baseline characteristics were investigated for association with trial drop-out using logistic regression (Additional File 2).

Analyses of change in primary and secondary outcomes followed intention-to-treat principles. The three continuous primary outcome variables (minutes of MVPA, fruit serves, vegetable serves) were analysed using linear mixed models with fixed effects for treatment group, time, and group*time interaction. Within-subject correlation due to repeated time measurements was accounted for using a residual covariance structure of compound symmetry, based on comparison of Akaike’s Information Criterion (AIC) for alternative structures. For each outcome variable, the following potential confounding variables were tested for three-way interactions (group*time*variable): age (categorised), gender identity, primary mental health diagnosis (categorised as per Table 1), and use of antipsychotic medications with known metabolic risk (Yes/No) [47]. The same analyses were conducted for continuous secondary outcomes (weight, BMI).

Logistic regression was conducted using generalised linear mixed models for the dichotomous primary and secondary outcomes (‘attempts to change’ variables). For the linear mixed models, residual covariance structures were used to address correlation due to repeated measurements.

An exploratory per-protocol analysis was undertaken as above, restricting the intervention group to participants who had enrolled in GHS coaching and completed at least one coaching call (enrolees), defined by GHS data.

### Sample size and power

To accommodate for the anticipated rates of service enrolment and completion within the intervention group, the trial protocol specified recruitment of 675 participants with a 4:1 intervention-to-control ratio (540 intervention, 135 control), estimating 55% would provide follow-up data (*n* = 297 intervention, *n*= 75 control, total 372). For the intention-to-treat analyses this sample would provide an 80% power to detect a true difference between groups at follow-up of 0.58 vegetable serves per day (SD = 1.6), 0.47 fruit serves per day (SD = 1.3) and 109 min of moderate/vigorous physical activity per week (SD = 300) [32].

## Results

### Participant flow

Figure 1 summarises participant flow. In total, 684 participants completed the baseline survey and were randomised (intervention *n* = 549, control *n* = 135). Six months post-baseline, 525 (76.8%) participants were re-contacted and 398 (58.2%) completed the follow-up survey; 311/549 (56.6%) intervention and 87/135 (64.4%) control.

### Baseline data

Baseline characteristics were comparable across groups (Table 1). A range of primary mental health conditions were reported including depression, schizophrenia, and anxiety, and most were taking medication for their mental health condition. Trial drop-out was more likely for participants who were younger, most disadvantaged, and living in major cities (Additional File 2).

**Table 1:** Baseline characteristics

Characteristic	Intervention	Control
	(*n* = 549)	(*n* = 135)
	n, (%)	n, (%)
Demographic characteristics/recruitment method		
Recruitment method		
CMHS	371 (67.6)	87 (64.4)
Social media	94 (17.1)	24 (17.8)
Previous research participant (originally from CMHS)	84 (15.3)	24 (17.8)
Age (years)		
mean (SD)	40.70 (13.3)	41.86 (13.0)
median (range)	40 (18–75)	42 (18–72)
Age		
18–29	147 (26.8)	25 (18.5)
30–39	117 (21.3)	38 (28.2)
40–49	126 (23.0)	30 (22.2)
50–59	114 (20.8)	28 (20.7)
60 +	45 (8.2)	14 (10.4)
Gender identity		
Female	343 (62.5)	82 (60.7)
Male	200 (36.4)	50 (37.0)
Other	6 (1.1)	3 (2.2)
SEIFA category		
Most disadvantaged (below NSW median)	324 (59.0)	79 (58.5)
Least disadvantaged (median and above)	224 (40.8)	56 (41.5)
Remoteness		
Major Cities	414 (75.4)	105 (77.8)
Inner/outer regional or remote	134 (24.4)	30 (22.2)
Living arrangement		
Private housing	443 (80.7)	104 (77.0)
Public housing/accommodation	74 (13.5)	20 (14.8)
Short-stay accommodation	5 (0.9)	5 (3.7)
Temporarily staying with friends or family	11 (2.0)	1 (0.7)
Care or treatment facility	2 (0.4)	1 (0.7)
Other	14 (2.55)	3 (0.2)
Identifies as Aboriginal and/ or Torres Strait Islander		
Aboriginal and/or Torres Strait Islander	50 (9.1)	11 (8.2)
Neither	494 (90.0)	122 (90.4)
Don’t know/refused/missing	5 (0.9)	2 (1.5)
Employment status		
Full-time employment	79 (14.4)	10 (7.4)
Part-time/casual employment	113 (20.6)	30 (22.2)
Unable to work due to a medical condition	185 (33.7)	50 (37.0)
Retired	19 (3.5)	9 (6.7)
Not working – other	152 (27.69)	36 (26.7)
Highest education level achieved		
Less than school certificate	39 (7.10)	15 (11.1)
School certificate	101 (18.40)	22 (16.3)
Higher school certificate	79 (14.39)	11 (8.2)
TAFE or diploma	213 (38.80)	56 (41.5)
University undergraduate degree or higher	116 (21.13)	31 (23.0)
Clinical characteristics		
Primary mental health diagnosis		
Depression	119 (21.7)	35 (25.9)
Bipolar disorder	71 (12.9)	21 (15.6)
Schizophrenia	105 (19.1)	27 (20.0)
Psychosis	6 (1.1)	1 (0.7)
Anxiety	76 (13.8)	16 (11.9)
Substance Use	1 (0.2)	1 (0.7)
Personality disorder	48 (8.7)	10 (7.4)
Eating disorder	19 (3.5)	3 (2.2)
Other	81 (14.8)	15 (11.1)
Don’t know/prefer not to say	23 (4.2)	6 (4.4)
Count of mental health medications taken		
0	84 (15.4)	16 (11.8)
1	168 (29.3)	46 (34.1)
2 +	195 (35.5)	45 (33.3)
Don’t know/prefer not to say	100 (18.6)	28 (20.7)
Taking antipsychotic medication (metabolic risk category^a^)		
Not taking medication for mental health condition	86 (19.2)	16 (15.0)
No/Unknown metabolic risk	164 (36.6)	43 (40.2)
Yes—low metabolic risk	26 (5.8)	8 (7.5)
Yes—moderate metabolic risk	91 (20.3)	27 (25.2)
Yes—high metabolic risk	81 (18.1)	13 (12.1)
BMI categories^b^		
Underweight (BMI < 18.5)	8 (1.5)	4 (3.0)
Healthy weight (18.5 ≤ BMI < 25)	100 (18.2)	18 (13.3)
Overweight (25 ≤ BMI < 30)	107 (19.5)	32 (23.7)
Obese (30 ≤ BMI < 40)	154 (28.1)	40 (29.6)
Morbidly obese (BMI ≥ 40)	59 (10.8)	12 (8.9)
Alcohol consumption (AUDIT-C), *mean (SD)*^c^	2.83 (3.4)	2.69 (3.2)
Non-drinker (never consumes alcohol)	186 (33.9)	50 (37.0)
Low risk	181 (33.0)	41 (30.4)
Moderate	95 (17.3)	25 (18.5)
High risk	58 (10.6)	13 (9.6)
Severe risk/Dependence	15 (2.7)	1 (0.7)
Sedentary behaviour – hrs/day, *mean (SD)*^d^	12.44 (3.8)	12.40 (3.9)
Psychological wellbeing (K6)		
Low mental distress (score < 5)	0 (0)	0 (0)
Moderate mental distress	167 (54.9)	45 (60.0)
Severe mental distress	137 (45.0)	30 (40.0)
Quality of life (AQoL-4D), mean (SD)		
AQoL-4D utility score	0.41 (0.3)	0.40 (0.3)
Independent living dimension score	0.90 (0.2)	0.90 (0.2)
Relationships dimension score	0.60 (0.3)	0.60 (0.3)
Senses dimension score	0.93 (0.1)	0.93 (0.1)
Mental health dimension score	0.68 (0.2)	0.67 (0.2)
Health risk behaviours (at risk)^e^		
Physical inactivity	214 (39.0)	56 (41.5)
Poor nutrition (fruit and/or vegetables)	527 (96.0)	133 (98.5)
Harmful alcohol consumption	168 (30.6)	39 (28.9)
Tobacco smoking	210 (38.3)	50 (37.0)

Some percentages do not add up to 100% due to missing data or condensed response options

^a^De Hert, M., Detraux, J., van Winkel, R., Yu, W., & Correll, C.Y. (2012). Metabolic and cardiovascular adverse effects associated with antipsychotic drugs. Nat. Rev. Endocrinol. 8, 114–126

^b^Calculated for participants providing self-reported weight and height. Excluded 19 intervention and 3 control participants with a primary mental health diagnosis of eating disorder

^c^Reports alcohol consumption within the past month. AUDIT-C uses 3 categorical items measuring levels of harmful alcohol consumption, each scored 0–4. Risk category scores are: low risk (males 0–4; females 0–3), medium risk (males 5–7; females 4–7), high risk (8–10), severe risk indicative of dependence (11–12)

^d^Average hours of sedentary activity per day calculated using the SIMPAQ alternate method for sedentary activity

^e^Health behaviour risk status was calculated as per Australian guidelines with ‘at risk’ defined as: physical activity (< 150 min per week of MVPA) [2]; consuming less than two serves of fruit and five serves of vegetables daily [48]; alcohol consumption (consumed > 2 standard drinks on an average day or > 4 standard drinks on any one occasion in the last month) [1, 38, 39, 49]; and tobacco smoking (any in the last month)

### Intervention fidelity

**Fig. 2:**
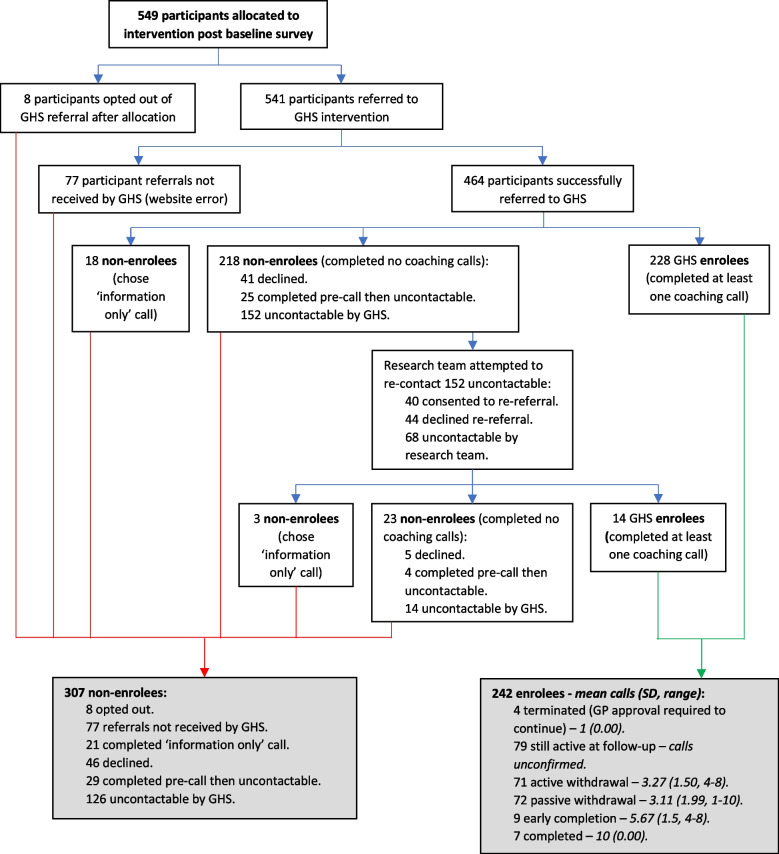
Intervention engagement flow diagram

All control participants were sent the information pack. Figure 2 summarises intervention group contact with GHS. After randomisation, eight participants opted out of GHS referral but did not withdraw from the study. The research team referred 541 participants to GHS. Due to a technical error with the GHS referral website, 77 referrals made after 8 October 2020 were not received by GHS and, as such, 464 referrals (85.8%) were successfully received. A total of 242 participants enrolled and completed at least one coaching call (‘enrolees’) (44% of those randomised to intervention group, 52% of received referrals; enrolling a mean of 23.8 days after referral (SD = 20.54, range 1–166, median = 19). Most enrolled in the standard program (*n* = 135/242, 56%), and a weight-related goal was most common (*n* = 135/242, 56%) (Additional File 3). The remaining 307 ‘non-enrolees’ did not complete any coaching calls.

The final program status at follow-up (six months post-recruitment) was identified for each enrolee (Fig. 2): 16 participants had completed the coaching program, 147 had withdrawn/terminated, and 79 were still actively engaged with the program. The 16 participants to complete within the six-month intervention period represent 2.9% of those allocated to the intervention group (and included in the intention-to-treat analysis), and 6.6% of enrolees.

The mean coaching calls completed within the six-month period (by final status) is reported in Fig. 2 for the 163 enrolees who had ended their contact with the GHS by follow-up. For those still active in the program, it was not possible to determine how many calls were completed within the intervention period.

### Outcomes

#### Intention-to-treat analyses

Results of the intention-to-treat analyses (all participants randomised) are in Table 2 (primary outcomes) and 3 (secondary outcomes). As no statistically significant three-way interactions were found when testing for potential confounders, the results from unadjusted models are presented. The difference in change (baseline to six-month follow-up) between intervention and control groups was not significant for any primary or secondary outcomes. There were small non-significant effects favouring the intervention group for weight, BMI (unadjusted differences Table 3), and for the ‘attempts to change’ variables. Non-significant effects for physical activity favour the intervention group when all participants are included, but not when those with extreme values are excluded (Table 2). Unadjusted differences for fruit and vegetable variables were very small.

**Table 2:** Intention-to-treat analysis – primary outcomes

	Get Healthy Service Intervention				Control					
Continuous outcomes	Baseline mean (SD)	n^a^	6 months Mean (SD)	n^a^	Baseline mean (SD)	N	6 months mean (SD)	N	Unadjusted difference (95% CI) at 6 months^c^	*p*-value
MVPA/week (all valid data)	450.4 (575.4)	517	474.8 (619.7)	303	459.6 (752.1)	125	386.0 (551.8)	83	94.14 (−81.89, 270.17)	.29
MVPA/week (excluding > 2.5 SD)^f^	396.8 (332.2)	400	433.5 (356.5)	243	363.2 (312.5)	92	410.9 (303.0)	62	−8.97 (−116.80, 98.87)	.87
Serves fruit/day	1.25 (1.26)	543	1.34 (1.11)	309	1.03 (1.10)	135	1.14 (1.14)	87	−0.12 (−0.41, 0.18)	.44
Serves vegetables/day	2.13 (1.48)	544	2.40 (1.50)	308	2.06 (1.70)	134	2.30 (1.65)	86	−0.05 (−0.41, 0.31)	.78
Dichotomous outcomes	Baseline n/N (%)		6 months n/N (%)		Baseline n/N (%)		6 months n/N (%)		Unadjusted ratio of odds ratios (95% CI)^d^	Unadjusted risk difference (%) (95% CI)^e^
Any attempts to change^b^	492/549 (89.6)		295/311 (94.9)		121/135 (89.6)		79/87 (90.8)		1.64 (0.61, 4.46) *p* = .33	3.18 (−4.65, 11.01) *p* = .43

^a^Includes 77 participants whose referral was not received by GHS

^b^Composite outcome including any attempt to change any of the following behaviours or weight: to increase physical activity, fruit consumption, or vegetable consumption; to make any other dietary changes; to lose weight (those with primary diagnosis eating disorder excluded from weight attempts analysis); or to reduce alcohol consumption (those reporting no alcohol consumption excluded from ‘attempts to reduce alcohol consumption’ analysis)

^c^Differences of means Intervention (six months – baseline) – Control (six months-baseline)

^d^OR (Intervention (six months/baseline))/OR (Control six months/baseline))

^e^Differences of mean % Intervention (six months – baseline) – Control (six months-baseline)

^f^As recommended by Rosenbaum et al. [35] in validation of SIMPAQ for mental health population

**Table 3:** Intention-to-treat analysis – secondary outcomes

	Get Healthy Service Intervention				Control					
Continuous outcomes	Baseline mean (SD)	N	6 months Mean (SD)	n	Baseline mean (SD)	N	6 months mean (SD)	n	Unadjusted difference (95% CI) at 6 months^c^	*p*-value
Self-reported weight^a,^^b^	91.55 (24.74)	502	91.16 (23.61)	285	90.58 (24.82)	126	91.75 (24.31)	80	−1.33 (−3.22, 0.57)	.17
BMI^a,^^b^	31.73 (7.94)	488	31.37 (7.25)	274	31.02 (8.10)	123	31.30 (7.52)	77	−0.34 (−0.99, 0.31)	.31
Dichotomous outcomes	Baseline n/N (%)		6 months n/N (%)		Baseline n/N (%)		6 months n/N (%)		Unadjusted ratio of odds ratios (95% CI)^d^	Unadjusted risk difference (%) (95% CI)^e^
Attempts made to:										
increase physical activity	336/548 (61.3)		207/311 (66.6)		82/135 (60.7)		49/87 (56.3)		1.43 (0.81, 2.54) *p* = .22	8.47 (−5.26, 22.19) *p* = .23
increase fruit consumption	215/549 (39.2)		143/308 (46.4)		62/134 (46.3)		41/87 (47.1)		1.23 (0.71, 2.13) *p* = .45	5.06 (−8.47, 18.59) *p* = .46
increase vegetable consumption	231/548 (42.2)		175/310 (56.5)		59/133 (44.4)		39/86 (45.4)		1.64 (0.94, 2.84) *p* = .08	12.26 (−1.37, 25.88) *p* = .08
make any other dietary change	331/549 (60.3)		225/310 (72.6)		81/134 (60.5)		57/87 (65.5)		1.36 (0.76, 2.44) *p* = .30	6.54 (−6.69, 19.77) *p* = .33
lose weight^b^	303/530 (57.2)		193/298 (64.8)		67/132 (50.8)		49/84 (58.3)		0.95 (0.56, 1.62) *p* = .85	−1.52 (−14.42, 11.39) *p* = .82
reduce alcohol consumption	142/360 (39.4)		86/204 (42.2)		30/84 (35.7)		18/60 (30.0)		1.33 (0.71, 2.51) *p* = .37	6.49 (−7.52, 20.50) *p* = .36

^a^Calculated using self-reported weight and height

^b^Excluded 19 intervention and 3 control with a primary mental health diagnosis of eating disorder

^c^Differences of means Intervention (six months – baseline) – Control (six months-baseline)

^d^OR (Intervention (six months/baseline))/OR (Control six months/baseline))

^e^Differences of mean % Intervention (six months – baseline) – Control (six months-baseline)

#### Exploratory per-protocol analyses

Results comparing the control group with the 242 enrolees (enrolee analysis) are in Tables 4 and 5. There were no statistically significant differences found for the health behaviour change variables, however there were significant differential increases in favour of the intervention group for the proportion of participants who made change attempts for: any health behaviour or weight, physical activity, vegetable consumption, other dietary changes. There were some small non-significant effects favouring the intervention group similar to those for the intention to treat analyses (unadjusted differences Tables 4, 5).

**Table 4:** Per-protocol: enrolee analysis – primary outcomes

	Get Healthy Service Intervention				Control					
Continuous outcomes	Baseline mean (SD)	N	6 months Mean (SD)	n	Baseline mean (SD)	n	6 months mean (SD)	n	Unadjusted difference (95% CI) at 6 months^c^	*p*-value
MVPA/week (all valid data)	405.2 (524.9)	235	477.2 (582.4)	158	459.6 (752.1)	125	386.0 (551.8)	83	130.20 (−48.43, 308.83)	.15
MVPA/week (excluding > 2.5 SD)^a^	355.7 (304.0)	188	468.2 (369.1)	125	363.2 (312.5)	92	410.9 (303.0)	62	49.95 (−60.66, 160.57)	.37
Serves fruit/day	1.24 (1.33)	240	1.47 (1.07)	161	1.03 (1.10)	135	1.14 (1.14)	87	0.04 (−0.31, 0.38)	.84
Serves vegetables/day	2.09 (1.44)	240	2.49 (1.42)	162	2.06 (1.70)	134	2.30 (1.65)	86	0.08 (−0.34, 0.50)	.71
Dichotomous outcomes	Baseline n/N (%)		6 months n/N (%)		Baseline n/N (%)		6 months n/N (%)		Unadjusted ratio of odds ratios (95% CI)^d^	Unadjusted risk difference (%) (95% CI)^e^
Any attempts to change^b^	217/242 (89.7)		158/162 (97.5)		121/135 (89.6)		79/87 (90.8)		3.68 (1.03, 13.23) *p* = *.05*	6.08 (−2.29, 14.44) *p* = *.15*

^a^As recommended by Rosenbaum et al. [35] in validation of SIMPAQ for mental health population

^b^Composite outcome including any attempt to change any of the following behaviours or weight: to increase physical activity, fruit consumption, or vegetable consumption; to make any other dietary changes; to lose weight (those with primary diagnosis eating disorder excluded from weight attempts analysis); or to reduce alcohol consumption (those reporting no alcohol consumption excluded from ‘attempts to reduce alcohol consumption’ analysis)

^c^Differences of means Intervention (six months – baseline) – Control (six months-baseline)

^d^OR (Intervention (six months/baseline))/OR (Control six months/baseline))

^e^Differences of mean % Intervention (six months – baseline) – Control (six months-baseline)

**Table 5:** Per-protocol: enrolee analysis – secondary outcomes

	Get Healthy Service Intervention				Control					
Continuous outcomes	Baseline mean (SD)	N	6 months Mean (SD)	n	Baseline mean (SD)	n	6 months mean (SD)	n	Unadjusted difference (95% CI) at 6 months^c^	*p*-value
Self-reported weight^a,^^b^	92.94 (25.57)	222	92.46 (24.43)	147	90.58 (24.82)	126	91.75 (24.31)	80	−1.24 (−3.11, 0.63)	.19
BMI^a,b^	32.22 (7.95)	218	32.09 (7.62)	142	31.15 (8.10)	123	31.30 (7.52)	77	−0.37 (−1.04, 0.26)	.27
Dichotomous outcomes	Baseline n/N (%)		6 months n/N (%)		Baseline n/N (%)	6 months n/N (%)			Unadjusted ratio of odds ratios (95% CI)^d^	Unadjusted risk difference (%) (95% CI)^e^
Attempts made to:										
increase physical activity	144/241 (59.8)		121/162 (74.7)		82/135 (60.7)	49/87 (56.3)			2.23 (1.16, 4.27) *p* = .02**	17.88 (2.89, 32.88) *p* = .02**
increase fruit consumption	98/242 (40.5)		82/159 (51.6)		62/134 (46.3)	41/87 (47.1)			1.43 (0.78, 2.64) p = .25	8.85 (−6.30, 24.01) p = .25
increase vegetable consumption	103/241 (42.7)		101/161 (62.7)		59/133 (44.4)	39/86 (45.4)			2.07 (1.11, 3.86) *p* = .02**	17.87 (2.61, 33.13) *p* = .02**
make any other dietary change	149/242 (61.6)		137/161 (85.1)		81/134 (60.5)	57/87 (65.5)			2.71 (1.38, 5.30) *p* = .004**	17.53 (3.89, 31.16) *p* = .012**
lose weight^b^	134/233 (57.5)		114/154 (74.0)		67/132 (50.8)	49/84 (58.3)			1.42 (0.76, 2.66) *p* = *.27*	7.10 (−7.50, 21.71) *p* = *.34*
reduce alcohol consumption	60/154 (39.0)		45/105 (42.9)		30/84 (35.7)	18/60 (30.0)			1.35 (0.68, 2.66) *p* = *.39*	6.75 (−8.53, 22.02) *p* = *.38*

^a^Calculated using self-reported weight and height

^b^Excluded those with a primary mental health diagnosis of eating disorder

^c^Differences of means Intervention (six months – baseline) – Control (six months-baseline)

^d^OR (Intervention (six months/baseline))/OR (Control six months/baseline))

^e^Differences of mean % Intervention (six months – baseline) – Control (six months-baseline)

^**^p < .05

#### Acceptability and appropriateness of GHS to enrolees

The AIM and IAM scores, by final status, are in Table 6.

**Table 6:** Acceptability and appropriateness of GHS for enrolees, by program status 6 months from baseline

Measure	Intervention		
	Withdrawal (active/passive)	Completed (incl. early)	Still active
	(*n* = 73)	(*n* = 15)	(*n* = 72)
	mean, (SD)	mean, (SD)	mean, (SD)
Acceptability (AIM) total (range 4–20)^a^	16.56 (3.71)	17.71 (2.84)	18.03 (2.89)
The GHS met my approval	4.19 (1.05)	4.43 (0.76)	4.54 (0.75)
The GHS was appealing to me	4.13 (1.08)	4.36 (0.74)	4.37 (0.86)
I liked the GHS	4.08 (1.05)	4.43 (0.76)	4.53 (0.85)
I welcomed the GHS	4.16 (1.03)	4.50 (0.65)	4.60 (0.76)
Appropriateness (IAM) total (range 4–20)	16.01 (4.44)	17.64 (2.53)	17.80 (3.20)
The GHS is fitting	3.98 (1.16)	4.36 (0.74)	4.39 (0.89)
The GHS is suitable	4.08 (1.13)	4.43 (0.65)	4.50 (0.80)
The GHS is applicable	4.11 (1.18)	4.43 (0.65)	4.49 (0.79)
The GHS is a good match	3.84 (1.29)	4.43 (0.65)	4.35 (0.94)

These measures were collected in the follow-up survey. As the survey completion rate was lower for those who had withdrawn from the program (67%) than for those still active (91%) or who had completed the program, the results may be less representative of the withdrawal group

^a^Items scored on Likert scale from 1 (strongly disagree) to 5 (strongly agree). Higher scores indicate greater acceptability or appropriateness

## Discussion

This is the first randomised controlled trial to evaluate the effectiveness of referral to a population-level telephone coaching service to support health behaviour change in healthy eating, physical activity, or weight management in people with a mental health condition. Intention-to-treat analyses found referral to the GHS by the research team did not lead to statistically significant differential change compared to the control group for either primary or secondary outcomes examined six months post referral. Per-protocol exploration comparing the control group to intervention enrolees found no significant difference in health behaviour change, weight, or BMI, but enrolees were significantly more likely to attempt to: change a health behaviour/weight, increase their physical activity, increase vegetable consumption, and make other dietary changes. Small non-significant effects for weight and BMI favoured the intervention group.

The findings should be considered within the context of limited exposure of participants to the coaching program, with just over half of intervention participants not having completed any coaching calls, and very few having completed the program at follow-up; limiting opportunity to detect significant improvements. The anticipated enrolment rate (50%) [32] and the number of enrolees providing follow-up data were reached. However, we had anticipated that more enrolees would have completed the program within the six-months.

The trial commenced just prior to the emergence of COVID-19: approximately 15% of participants had been recruited when ‘stay at home’ restrictions were introduced in NSW [50]. GHS reported minimal disruption to operations, including numbers of referrals and enrolments, during the study period. However, all participants experienced the public health restrictions imposed during this time. We are unsure how these factors may have created behaviour change challenges for participants [51, 52], or impacted intervention engagement and trial results.

Our results for behaviour and weight change are inconsistent with previous work evaluating the impact of telephone coaching for mental health participants, which generally found small/moderate yet significant improvements in at least one outcome [26–28, 53]. Two previous evaluations, including one of GHS participants with a mental health condition [30] and one of a 12-month South African telephone weight management program specifically for those with serious mental illness referred by psychiatrists [26], have reported small to moderate significant positive effects on outcomes including weight and BMI [26, 30] fruit intake, vegetable intake and physical activity [30]. The average calls completed by participants in both these studies were likely higher than the current trial (Bradley et al. 10.7 calls for completers, 6.3 for early completers), and their outcome analyses were conducted on those who had completed the coaching programs, which potentially contributed to the more positive findings. It is also possible that telephone coaching programs designed for delivery to people with a mental health condition may have different effects and perhaps lower attrition than those available to the general population. The magnitude of the significant within-group effects for weight and BMI reported in the Bradley et al. evaluation of the GHS were −1.46 kg (− 2.32 to − 0.59), and − 0.63 BMI points (− 0.96 to − 0.30) respectively [30], while Temmingh et al. [26] reported a mean weight loss for the completer group of −4.8 kg (95% CI, −5.67 to −3.82). In Temmingh et al.’s study, 61.4% of referrals completed the 12-month program (compared with 31% completing the GHS in Bradley et al.).

The findings suggest that program enrolment increased the proportion attempting behaviour change by around 20% more in the intervention group than control for each of three behaviours. In some models of behaviour change ‘readiness to change’ and recent change attempts are considered precursors to successful long-term change [54]. Within smoking research, ‘quit attempts’ are reported as a known precursor to quitting [55, 56], however, attempts to change are rarely reported for lifestyle interventions for other health behaviours. We are unsure what value, if any, increasing attempts to change may hold for participants, however, this measure could indicate some positive effects for program enrolees.

There are several strengths and additional limitations. Strengths include use of a randomised controlled design and outcome data collected by blinded research staff [30]. In addition to the limited number of intervention participants to have completed the program by follow-up, we note the limitations of self-report data, although objective means of measuring weight and physical activity were not feasible. For the physical activity outcome, the study was powered to detect a large effect size, and there was large variability for the confidence intervals for effects, suggesting caution in interpreting non-significant effects for this outcome. Fruit and vegetable consumption, while a key indicator of a healthy diet, may not be the most salient dietary change for this population, as reflected in the high proportion of participants who reported attempts to change other dietary aspects. A significant difference in trial drop-out within the intervention group was also observed; those who enrolled in GHS were less likely to be lost to trial attrition than non-enrolees, a potential bias in estimating the differences between groups. This trial also assessed for change in variables irrespective of change goal identified by the participant. For example, 56% of enrolees (*n* = 135) set a weight-related goal yet change in weight was assessed for all participants as a comparative control subgroup could not be identified.

Further research could focus on better understanding the experience of people with a mental health condition in programs like GHS designed for the general population. Qualitative interviews with GHS coaches about their experience of coaching clients with mental health conditions have suggested that such clients may face additional challenges with behaviour change but also that some benefits may be derived more broadly, such as preparing for or attempting to change or improvements in mental health [57]. In the current trial, acceptability and appropriateness scores were reasonably high, and higher for those still active in coaching or who had completed, suggesting the program was viewed positively. A qualitative exploration of trial participants’ experience with the program is underway. Future qualitative research would provide an in-depth understanding of reasons for non-completion, and could inform strategies to improve rates of completion.

Exploring strategies to improve program engagement may also be of benefit. The strategy used to boost conversion of referrals to enrolments in this study, whereby the researchers attempted to recontact those the GHS could not reach after referral, resulted in a small proportion (9%) enrolling. This suggests that additional contact attempts and/or progress check-ins from the referrer may slightly improve conversion to program enrolment. Identifying more effective strategies to convert referrals into enrolment, and exploring predictors of enrolment following referral, and program completion following enrolment, may be of benefit to referring practitioners to help identify patients who may require more support to engage with the program beyond referral.

## Conclusions

Referral of people with a mental health condition to a population telephone lifestyle coaching service did not significantly change physical activity, fruit or vegetable intake, weight, or BMI after 6 months, when compared with a control group receiving mailed information, although there were small non-significant effects for weight and BMI. The number of coaching calls completed by the intervention group by follow-up was low, many of those referred did not enrol, and many of those who did enrol withdrew from or were still engaged in the program when due for follow-up. Enrolment in coaching did support behaviour change attempts and the program was rated as acceptable and appropriate. Additional time to complete the program and additional support to maintain engagement may have been required to allow more robust testing of the study aims.

## Supplementary Information

Additional file 1.

Additional file 2.

Additional file 3.

## Data Availability

Deidentified datasets used and/or analysed during the current study are available from the corresponding author upon reasonable request using a secure data transfer link.

## Abbreviations

CATI: Computer assisted telephone interview
RCT: Randomised controlled trial
NSW: New South Wales (Australia)
GHS: The NSW Get Healthy Coaching and Information Service GHS
BMI: Body mass index
MVPA: Moderate-vigorous physical activity
