# Supplementary material for: Effectiveness of referral to a population-level telephone coaching service for improving health risk behaviours in people with a mental health condition: a randomised controlled trial
Source: BMC Public Health. 2025 Feb 19;25:677. doi: 10.1186/s12889-025-21614-w (PMC11837387; doi:10.1186/s12889-025-21614-w)
Supplement: Supplementary file 1 — Additional file 1. [file 12889_2025_21614_MOESM1_ESM.docx]

Additional file 1: Survey Schedule for reported items

**{Baseline only} What is your date of birth: __________________________________**

{Baseline only} Age: {calculated}

**{Baseline only} What is your gender identity?**

- Female
- Male
- Other
- Don't know
- Refused/Prefer not to say

Nutrition

The following questions are designed to get a better understanding of some aspects of your health and possible interest in making some lifestyle changes. Please answer the questions as honestly and accurately as you can. If there are any questions you don't want to answer, you can respond with "I'd prefer not to say".

The first few questions are about your eating habits over the last month.

We understand this may have been impacted by the recent changes due to the coronavirus, but for the sake of these questions, please think about your typical behaviours over the last month.

**A serve is one medium piece (about the size of your fist) or two small pieces of fruit (combined to about the size of your fist), or 1 cup of diced pieces. This includes fresh, dried, frozen or tinned fruit. How many serves of fruit do you eat in a usual day? __________________________________**

**Have you attempted to increase your fruit consumption in the last four months?**

- Yes
- No
- Don't know
- Refused/prefer not to say

**A serve is half a cup of cooked vegetables, dried or canned peas, beans, or lentils, (one cupped hand) or one cup of salad vegetables (two cupped hands). This can include fresh, dried, frozen or tinned vegetables. How many serves of vegetables do you eat in a usual day? __________________________________**

**Have you attempted to increase your vegetable consumption in the last four months?**

- Yes
- No
- Don't know
- Refused/prefer not to say

**Have you attempted to make any other changes to your diet or nutrition in the last four months?**

- Yes
- No
- Don't know
- Refused/prefer not to say

Physical Activity

I am now going to ask you about what you have been doing over the past seven days, including time spent in bed, sitting or lying down, walking, exercise, sport, and other activities.

**What time did you mostly go to bed over the past seven days? __________________________________ (24HR TIME)**

**What time did you mostly get out of bed over the past seven days? __________________________________ (24HR TIME)**

Average hours in bed per night: {calculated}

Hours remaining: {calculated}

**That leaves approximately {calculated} hours a day for other activities. What days in the past seven days did you walk for exercise or recreation or to get to or from places?**

- Monday
- Tuesday
- Wednesday
- Thursday
- Friday
- Saturday
- Sunday
- Don't know
- Refused/prefer not to say

**{If checked} How many minutes did you usually spend walking on {DAY}? __________________________________**

INTERVIEWER NOTE: Answer in total number of minutes

Average hours walking per day: {calculated}

**Now think about any activity that you do for exercise and sport, such as jogging, running, swimming, bike riding, going to the gym, yoga, etc. Which days in the past week did you do any of these, or similar activities?**

- Monday
- Tuesday
- Wednesday
- Thursday
- Friday
- Saturday
- Sunday
- Don't know
- Refused/prefer not to say

**{If checked} What activities did you do on {DAY}? __________________________________**

**And how many minutes did you spend doing that activity? __________________________________**

INTERVIEWER NOTE: List total number of minutes of all activities

Total hours of sport or exercise: {calculated}

Average hours of sport or exercise per day: {calculated}

**Now think about any other physical activities that you did as part of your work, or activities you did while at home such as gardening or household chores. How many minutes did you spend on these activities on most days? __________________________________**

INTERVIEWER NOTE: This does not include walking, sport or exercise. Answer in total number of minutes.

Average hours sedentary per day: {calculated}

**That leaves approximately {calculated} hours a day for activities where you were sitting or lying down, such as when you are eating, reading, watching TV or using electronic devices. This time would also include if you are sitting at work, during transport, leisure time, etc. Are there any other activities you think we may have missed?**

- Yes
- No
- Don't know
- Refused/prefer not to say

**{If YES checked} What activity have we missed?**

INTERVIEWER NOTE: Go back and enter new activity missed above

**Out of these {calculated} hours, how much of this time is spent napping? __________________________________**

INTERVIEWER NOTE: Answer in total number of minutes

**Have you attempted to increase your physical activity in the last four months?**

- Yes
- No
- Don't know
- Refused/prefer not to say

**Have you made any attempts to lose weight in the last four months?**

- Yes
- No
- Don't know
- Refused/prefer not to say

Tobacco, Alcohol, Height and Weight

The next few questions are about smoking and alcohol intake over the last month as well as your height and weight.

**In the last month, did you smoke any tobacco products?**

- Yes
- No {skip next question}
- Don't know
- Refused/prefer not to say

**How often did you smoke tobacco products in the last month?**

- Less than once a week
- At least once a week
- Daily
- Don't know
- Refused/prefer not to say

**Have you previously smoked?**

- No, never smoked {skip next question}
- I have smoked but never regularly
- Yes, but quit 6 or more months ago
- Yes, but quit less than 6 months ago
- Other: Please specify__________________________________
- Don't know
- Refused/prefer not to say

**Have you attempted to reduce or quit smoking in the last four months?**

- Yes
- No
- Don't know
- Refused/prefer not to say

**In the last month, how often have you had a drink containing alcohol?**

- Never - I don't drink {skip to current weight}
- None in the last month
- Once
- 2 to 4 times a month
- 3 times a week
- 4 or more times a week
- Don't know
- Refused/prefer not to say

**How many standard drinks would you have on a typical drinking day? A standard drink is 1 schooner of light beer, 1 middy of full-strength beer, one 100ml glass of wine or one 30ml nip of spirits)_____________________________**

**How often would you have more than four standard drinks on one occasion?**

- Never
- Less than monthly
- Monthly
- Weekly
- Daily or almost daily
- Don't know
- Refused/prefer not to say

**Have you attempted to reduce your alcohol consumption in the last four months?**

- Yes
- No
- Don't know
- Refused/prefer not to say

**What is your current weight? __________________________________**

INTERVIEWER NOTE: Select unit of measurement and open field(s) will appear.

- Kilograms: **__________________________________**
- Pounds: **__________________________________**
- Stone: **__________________________________**
- Don't know
- Refused/prefer not to say

**{Baseline only} What is your current height? __________________________________**

INTERVIEWER NOTE: Select unit of measurement and open field(s) will appear.

- Centimetres: **__________________________________**
- Feet and inches: **__________________________________**
- Don't know
- Refused/prefer not to say

Psychological Wellbeing - K6

This next section of questions is about your mental wellbeing and how you have been feeling over the last month. If you find any of these questions distressing, please let me know. I'll remind you, you can always choose not to provide an answer for any question.

Thinking about the LAST 30 DAYS, the following questions ask about how you have been feeling during that time. For each question, please provide an answer between 1 - None of the time, and 5 - All of the time.

During the past 30 days, about how often did you feel...

|  | 1 - None of the time | 2 | 3 | 4 | 5 – All of the time | Don’t know | Refused |
| --- | --- | --- | --- | --- | --- | --- | --- |
| Nervous: | ○ | ○ | ○ | ○ | ○ | ○ | ○ |
| Hopeless: | ○ | ○ | ○ | ○ | ○ | ○ | ○ |
| Restless or fidgety: | ○ | ○ | ○ | ○ | ○ | ○ | ○ |
| So depressed that nothing could cheer you up: | ○ | ○ | ○ | ○ | ○ | ○ | ○ |
| That everything was an effort: | ○ | ○ | ○ | ○ | ○ | ○ | ○ |
| Worthless: | ○ | ○ | ○ | ○ | ○ | ○ | ○ |

**The last six questions asked about feelings that might have occurred during the past 30 days. Taking them altogether, did these feelings occur MORE OFTEN in the past 30 days than is usual for you, ABOUT THE SAME as usual or LESS OFTEN than usual?**

- More often than usual
- About the same as usual
- Less often than usual
- Don't know
- Refused/prefer not to say

**The next few questions are about how these feelings may have affected you in the past 30 days. __________________________________**

**During the past 30 days, how many days out of 30 were you totally unable to work or carry out your normal activities because of these feelings? __________________________________**

**NOT counting the days you reported in the previous question, how many days in the past 30 were you able to do only HALF OR LESS of what you would normally have been able to do, because of these feelings? __________________________________**

**During the past 30 days, how many times did you see a doctor or other health professional about these feelings? __________________________________**

**During the past 30 days, how often have physical health problems been the main cause of these feelings?**

- None of these times
- A little of these times
- Some of the time
- Most of the time
- All of the time
- Don't know
- Refused/prefer not to say

Quality of Life – AQoL(4D)

These next few questions are about independent living. Please think about the option that best describes your situation as it has been over the past week.

**Do you need any help looking after yourself? (For example: dressing, bathing, eating)**

INTERVIEWER NOTE: PLEASE READ OUT RESPONSES:

- I need no help at all
- I occasionally need some help with personal care tasks
- I need help with the more difficult personal care tasks
- I need daily help with most or all personal care tasks
- Don't know
- Refused/prefer not to say

**What about doing household tasks: (For example: cooking, cleaning the house, washing)**

INTERVIEWER NOTE: PLEASE READ OUT RESPONSES

- I need no help at all.
- Occasionally I need some help with household tasks.
- I need help with the more difficult household tasks.
- I need daily help with most or all household tasks .
- Don't know
- Refused/prefer not to say

**Thinking about how easily you can get around the home and community, would you say:**

INTERVIEWER NOTE: PLEASE READ OUT RESPONSES

- You get around your home and community by yourself without any difficulty.
- You find it difficult to get around your home and community by yourself.
- You cannot get around the community by yourself, but you can get around your home with some difficulty.
- You cannot get around either the community or your home by yourself.
- Don't know
- Refused/prefer not to say

These next few questions are about your relationships. Please think about the option that best describes your situation as it has been over the past week.

**Because of your health, your relationships (for example: with your friends, partner or parents) generally:**

INTERVIEWER NOTE: PLEASE READ OUT RESPONSES

- Are very close and warm.
- Are sometimes close and warm.
- Are seldom close and warm.
- You have no close and warm relationships.
- Don't know
- Refused/prefer not to say

**Thinking about your relationship with other people, would you say:**

INTERVIEWER NOTE: PLEASE READ OUT RESPONSES

- You have plenty of friends, and are never lonely.
- Although you have friends, you are occasionally lonely.
- You have some friends, but are often lonely for company.
- You are socially isolated and feel lonely.
- Don't know
- Refused/prefer not to say

**Thinking about your health and your relationship with your family, would you say:**

INTERVIEWER NOTE: PLEASE READ OUT RESPONSES

- Your role in the family is unaffected by your health.
- There are some parts of your family role you cannot carry out.
- There are many parts of your family role you cannot carry out.
- You cannot carry out any part of your family role.
- Don't know
- Refused/prefer not to say

These next few questions are about your seeing, hearing, and communication. Please think about the option that best describes your situation as it has been over the past week.

**Thinking about your vision, including when using your glasses or contact lenses if needed, would you say:**

INTERVIEWER NOTE: PLEASE READ OUT RESPONSES

- You see normally.
- You have some difficulty focusing on things, or you do not see them sharply. For example: small print, a newspaper or seeing objects in the distance.
- You have a lot of difficulty seeing things. Your vision is blurred. For example: You can see just enough to get by with.
- You only see general shapes, or are blind. For example: You need a guide to move around.
- Don't know
- Refused/prefer not to say

**Thinking about your hearing, including using your hearing aid if needed, would you say:**

INTERVIEWER NOTE: PLEASE READ OUT RESPONSES

- You hear normally.
- You have some difficulty hearing or you do not hear clearly. For example: You ask people to speak up, or turn up the TV or radio volume.
- You have difficulty hearing things clearly. For example: Often you do not understand what is said. You usually do not take part in conversations because you cannot hear what is said.
- You hear very little indeed. For example: You cannot fully understand loud voices speaking directly to you.
- Don't know
- Refused/prefer not to say

**When you communicate with others: (For example: by talking, listening, writing or signing) would you say:**

INTERVIEWER NOTE: PLEASE READ OUT RESPONSES

- You have no trouble speaking to them or understanding what they are saying.
- You have some difficulty being understood by people who do not know you. You have no trouble, understanding what others are saying to you.
- You are only understood by people who know you well. You have great trouble understanding what others are saying to you.
- You cannot adequately communicate with others.
- Don't know
- Refused/prefer not to say

These next few questions are about your mental health Please think about the option that best describes your situation as it has been over the past week.

**Thinking about how you sleep, would you say:**

INTERVIEWER NOTE: PLEASE READ OUT RESPONSES

- You are able to sleep without difficulty most of the time.
- Your sleep is interrupted some of the time, but you are usually able to go back to sleep without difficulty.
- Your sleep is interrupted most nights, but you are usually able to go back to sleep without difficulty.
- You sleep in short bursts only. You are awake most of the night.
- Don't know
- Refused/prefer not to say

**Thinking about how you generally feel, would you say:**

INTERVIEWER NOTE: PLEASE READ OUT RESPONSES

- You do not feel anxious, worried or depressed.
- You are slightly anxious, worried or depressed.
- You feel moderately anxious, worried or depressed.
- You are extremely anxious, worried or depressed.
- Don't know
- Refused/prefer not to say

**How much pain or discomfort do you experience:**

INTERVIEWER NOTE: PLEASE READ OUT RESPONSES

- None at all.
- You have moderate pain.
- You suffer from severe pain.
- You suffer unbearable pain.
- Don't know
- Refused/prefer not to say

Mental Health

**{Baseline only} What is the primary mental health condition for which you are seeking support from your mental health service?**

- Depression
- Bipolar Disorder
- Schizophrenia
- Psychosis
- Anxiety
- Substance Use
- Personality Disorder
- Eating Disorder
- Other- Please specify: __________________________________
- Don't know
- Refused/prefer not to say

**{Baseline only} Do you have any secondary mental health conditions?**

- Yes
- No
- Don't know
- Refused/prefer not to say

**{If YES checked} Please list the secondary mental health conditions for which you are seeking support: __________________________________**

**Are you currently taking any medications for your mental health condition?**

- Yes
- No
- Don't know
- Refused/prefer not to say

**{If YES checked} What medication(s) are you taking?**

INTERVIEWER NOTE: Brand Name (pharmaceutical name)

- Abilify (Aripiprazole)
- Clozaril (Clozapine)
- Eskalith (Lithium)
- Invega (Palperidone)
- Largactil (Chlorpromazine)
- Latuda (Lurasidone)
- Lithobid (Lithium)
- Risperdal (Risperidone)
- Sandoz (Valproate)
- Saphris (Asenapine)
- Serdolect and Serlect (Sertindole)
- Seroquel (Quetiapine)
- Solian (Amisulpride)
- Thorazine (Chlorpromazine)
- Ziprasidone (Geodon)
- Zyprexa (Olanzapine)
- Other: Please specify: __________________________________
- Don't know
- Refused/prefer not to say

Interest and Confidence in Lifestyle Changes {Baseline only}

**{Baseline only} On a scale of 1 to 10, with 1 being not at all interested and 10 being extremely interested, how INTERESTED are you in making changes to your:**

|  | 1 – Not at all | 2 | 3 | 4 | 5 | 6 | 7 | 8 | 9 | 10 – Extremely | Don’t know | Refused |
| --- | --- | --- | --- | --- | --- | --- | --- | --- | --- | --- | --- | --- |
| Physical activity: | ○ | ○ | ○ | ○ | ○ | ○ | ○ | ○ | ○ | ○ | ○ | ○ |
| Fruit and/or vegetable consumption: | ○ | ○ | ○ | ○ | ○ | ○ | ○ | ○ | ○ | ○ | ○ | ○ |
| Other changes to your nutrition: | ○ | ○ | ○ | ○ | ○ | ○ | ○ | ○ | ○ | ○ | ○ | ○ |
| Weight: | ○ | ○ | ○ | ○ | ○ | ○ | ○ | ○ | ○ | ○ | ○ | ○ |
| Alcohol consumption: | ○ | ○ | ○ | ○ | ○ | ○ | ○ | ○ | ○ | ○ | ○ | ○ |

**{Baseline only} On a scale of 1 to 10, with 1 being not at all confident and 10 being extremely confident, how CONFIDENT are you in making changes to your:**

|  | 1 – Not at all | 2 | 3 | 4 | 5 | 6 | 7 | 8 | 9 | 10 – Extremely | Don’t know | Refused |
| --- | --- | --- | --- | --- | --- | --- | --- | --- | --- | --- | --- | --- |
| Physical activity: | ○ | ○ | ○ | ○ | ○ | ○ | ○ | ○ | ○ | ○ | ○ | ○ |
| Fruit and/or vegetable consumption: | ○ | ○ | ○ | ○ | ○ | ○ | ○ | ○ | ○ | ○ | ○ | ○ |
| Other changes to your nutrition: | ○ | ○ | ○ | ○ | ○ | ○ | ○ | ○ | ○ | ○ | ○ | ○ |
| Weight: | ○ | ○ | ○ | ○ | ○ | ○ | ○ | ○ | ○ | ○ | ○ | ○ |
| Alcohol consumption: | ○ | ○ | ○ | ○ | ○ | ○ | ○ | ○ | ○ | ○ | ○ | ○ |

Demographics {Baseline only}

Lastly, we have a few background questions about you.

**{Baseline only} What is your current living arrangement?**

- Living in a privately owned dwelling
- Living in public housing/accommodation
- Short-stay accommodation (i.e. hostel/boarding house)
- In a care or treatment facility
- Temporarily staying with friends/family
- No current accommodation
- Other: Please specify: __________________________________
- Don't know
- Refused/prefer not to say

**{Baseline only} Are you living on your own, with family or a partner, or with others?**

- On your own
- With partner/spouse/family
- With others
- Don't know
- Refused/prefer not to say

**{Baseline only} Are you currently working?**

- Full time
- Part time
- Casual
- Not working
- Don't know
- Refused/prefer not to say

**{If NOT WORKING checked} Do any of the following apply?**

- Unemployed
- Home duties
- Carer
- Studying
- Retired
- Volunteering
- Unable to work due to a medical condition
- Other: Please specify: __________________________________
- Don't know
- Refused/prefer not to say

**{Baseline only} What is the highest level of education you have obtained?**

- Never attended school
- Some primary school
- Completed primary school
- Some high school
- School certificate, Intermediate, Yr 10, 4th Form
- Completed HSC, Leaving, Year 12 or 6th Form
- TAFE certificate or diploma
- University, CAE, Degree or higher
- Don't know
- Refused/prefer not to say

**{Baseline only} Do you identify as Aboriginal or Torres Strait Islander?**

- Aboriginal
- Torres Strait Islander
- Both
- Neither
- Don't know
- Refused/prefer not to say

Acceptability and Appropriateness of GHS

{Follow-up only} {Intervention group only}

**Were you contacted by the Get Healthy Service?**

- Yes
- No {skip remaining questions}
- Don't know {skip remaining questions}
- Refused/prefer not to say {skip remaining questions}

**Did you begin the coaching program?**

- Yes, completed the program (includes early completion).
- Yes, but withdrew from the program before reaching my goals.
- Yes, still going with the program.
- Yes, but chose the information only program. {skip remaining questions}
- No, I declined the referral. {skip remaining questions}
- Don't know {skip remaining questions}
- Refused/prefer not to say {skip remaining questions}

**The next few questions ask about how acceptable you feel it is for The Get Healthy Service to provide coaching support for health behaviour change. Please respond to these questions by answering with a number between 1 and 5, with 1 being strongly disagree and 5 being strongly agree.**

|  | 1 – Strongly disagree | 2 | 3 | 4 | 5 – Strongly agree | Don’t know | Refused |
| --- | --- | --- | --- | --- | --- | --- | --- |
| The GHS met my approval: | ○ | ○ | ○ | ○ | ○ | ○ | ○ |
| The GHS was appealing to me: | ○ | ○ | ○ | ○ | ○ | ○ | ○ |
| I liked the GHS: | ○ | ○ | ○ | ○ | ○ | ○ | ○ |
| I welcomed the GHS: | ○ | ○ | ○ | ○ | ○ | ○ | ○ |

**The next few questions ask about how APPROPRIATE you feel the Get Healthy Service is to provide you with coaching support for health behaviour change, still answering with a number between 1 and 5, with 1 being strongly disagree and 5 being strongly agree.**

|  | 1 – Strongly disagree | 2 | 3 | 4 | 5 – Strongly agree | Don’t know | Refused |
| --- | --- | --- | --- | --- | --- | --- | --- |
| The GHS is fitting: | ○ | ○ | ○ | ○ | ○ | ○ | ○ |
| The GHS is suitable: | ○ | ○ | ○ | ○ | ○ | ○ | ○ |
| The GHS is applicable: | ○ | ○ | ○ | ○ | ○ | ○ | ○ |
| The GHS is a good match: | ○ | ○ | ○ | ○ | ○ | ○ | ○ |
