# Supplementary material for: Effectiveness of referral to a population-level telephone coaching service for improving health risk behaviours in people with a mental health condition: a randomised controlled trial
Source: BMC Public Health. 2025 Feb 19;25:677. doi: 10.1186/s12889-025-21614-w (PMC11837387; doi:10.1186/s12889-025-21614-w)
Supplement: Supplementary file 2 — Additional file 2. [file 12889_2025_21614_MOESM2_ESM.docx]

Additional file 2: Trial drop-out

Logistic regression carried out to identify factors associated with trial drop-out (failure to complete follow-up survey) included group allocation, gender identity, age category, taking medication for their mental health condition (Yes/No), recruitment method (social media/CMHS), SEIFA category, remoteness category, and primary mental health diagnosis. The modelling found three variables to be significant: age category, SEIFA category, and remoteness category (group allocation was not significant but was included in modelling). Drop-out rates increased with participant age, at 2.13 times higher for those aged 18-39 that those 40 years or older (CI 1.55-2.91); were 1.42 times higher for people in most compared to least disadvantaged areas (CI 1.03-1.96); and were higher for participants living in major cities (OR 1.51, CI 1.04-2.20) than in regional or remote locations. There was also a difference in drop-out rate within intervention group; GHS enrolees were 2.15 times less likely to drop out of the trial than non-enrolees (CI 1.51-3.05).
