# Supplementary material for: Effectiveness of referral to a population-level telephone coaching service for improving health risk behaviours in people with a mental health condition: a randomised controlled trial
Source: BMC Public Health. 2025 Feb 19;25:677. doi: 10.1186/s12889-025-21614-w (PMC11837387; doi:10.1186/s12889-025-21614-w)
Supplement: Supplementary file 3 — Additional file 3. [file 12889_2025_21614_MOESM3_ESM.docx]

Additional File 3: Intervention program and primary health goal selection

|  | n | % of enrolees |
| --- | --- | --- |
| GHS program selected: |  |  |
| Standard GHS program | 135 | 56% |
| Type-II diabetes | 74 | 31% |
| Diabetes prevention | 7 | 3% |
| Aboriginal and Torres Strait Islanders | 8 | 3% |
| Diabetes prevention for Aboriginal and Torres Strait Islanders | 4 | 5% |
| Alcohol reduction | 4 | 2% |
| GHS in pregnancy | 1 | 0.4% |
| Missing | 9 | 4% |
| Primary health goal: |  |  |
| Weight related | 135 | 56% |
| Waist circumference related | 2 | 1% |
| Food related | 62 | 26% |
| Exercise related | 31 | 13% |
| Alcohol related | 3 | 1% |
| Missing | 9 | 4% |
